# A Silane Cross-Linked Cellulose-Based Separator for Long-Life Lithium Metal Batteries Application

**DOI:** 10.3390/polym17091203

**Published:** 2025-04-28

**Authors:** Jinghao Cui, Hongliang Meng, Wei Li

**Affiliations:** Guangxi Key Laboratory of Clean Pulp & Papermaking and Pollution Control, College of Light Industry and Food Engineering, Guangxi University, Nanning 530004, China; 2216301005@st.gxu.edu.cn (J.C.); 2005170318@st.gxu.edu.cn (H.M.)

**Keywords:** cellulose-based separators, lithium metal batteries, wet strength, cycle performance

## Abstract

Cellulose-based separators with good electrolyte wettability and thermal stability have attracted extensive attention in the area of lithium metal battery (LMB) applications. However, their low mechanical strength in an electrolyte has seriously hindered their cycling performance of assembled LMB. Herein, a silane-crosslinked propionylated cellulose-based separator (PBF-GPTMS) was prepared. The resulting separator exhibited high wet strength (18.7 MPa) and electrolyte uptake (312 wt%). Molecular simulation revealed that Young’s modulus of the silanized propionylated cellulose model was 14.64 GPa under EC/DMC/DEC conditions, which was higher than that of the propionylated cellulose model (6.89 GPa). In particular, the XPS spectra of the Li foil in the PBF-GPTMS-assembled battery after cycling suggested a lower amount of HF formed during cycling. Accordingly, the assembled Li/Separator/LiFePO_4_ cell showed excellent cycle performance with capacity retention of 94.5% after 300 cycles at 0.5 C and 93.6% after 160 cycles at 1 C, respectively. This idea would provide novel insights into the design of bio-based separators for long-life LMBs.

## 1. Introduction

Lithium metal batteries (LMBs) have been considered one of the next-generation rechargeable batteries due to their high theoretical capacity, low electrochemical potential, and gravimetric density [1]. Unfortunately, the growth of Li dendrites seriously deteriorates the stability of the Li depositing/stripping process, which may lead to a short cycle lifespan and even safety hazards of LMBs [2].

As a crucial component of an LMB, the separator not only isolates the anode and cathode to prevent short circuits of the battery but also provides channels for ion transport [3]. Its properties play an important role in the working life of batteries [4]. However, traditional polyolefin separators display poor wettability to a liquid electrolyte, which would cause non-uniform transportation of lithium ions and the generation of lithium dendrites [5]. In addition, the PE/PP separators are prone to shrinkage at high temperatures, resulting in the contact between anode and cathode and further thermal runaway [6].

Different from polyolefin separators, cellulose-based materials with outstanding electrolyte wettability and thermal stability show great potential application in the LMBs [7,8]. Neither the source of cellulose isolation nor the method of its isolation are guaranteed for successful use as a separator [9]. Furthermore, although cellulose-based materials are known to have high mechanical strength due to their abundant inter-/intra-hydrogen bonds in the dry state, the derived separator may suffer from serious strength weakening after soaking in the electrolyte. This phenomenon could be attributed to the swelling and softening behaviors of the separator caused by electrolytes, which would affect the cycle performance of the assembled battery [10].

In order to solve this issue, researchers have tried to enhance the wet strength of cellulose-based separators in liquid electrolytes. Xie [11] prepared the separator by casting of the lignosulfonate powder and bleached eucalyptus cellulose pulp in a mold. The wet tensile strength of the separator achieved approximately 22 MPa due to the formation of hydrogen bonding between the lignin and cellulose molecules. The bulk resistance of the separator seemed to be high and its cycle performance was not investigated. Lv [12] fabricated a cellulose-based separator by blending cellulose pulp and cellulose nanofibrils (CNFs) followed by filtration. The separator demonstrated a wet tensile strength of 28 MPa, and the assembled cell displayed a capacity retention of 91% after 100 cycles at 1 C. Furthermore, due to the covalent crosslinking between lignosulfonate–polyamide-epichlorohydrin complex (LPC) nanoparticles and CNFs, the resulting separator displayed a capacity retention of 92.2% after 160 cycles at 1 C [13]. 

To further improve the comprehensive properties of cellulose-based separators, the side reactions during cycling should be considered. According to the literature, the hydroxyl groups in the separators would react with Li^+^ ions, leading to deterioration in the interface stability as well as a reduction in the charge/discharge efficiency [14]. Furthermore, the reaction between the residual trace water in the cellulose-based separators and LiPF_6_ may cause the formation of HF, which would impair the stability of separators and SEI layers to make their cycle life degrade [15,16].

Based on the above discussion, in this work, a novel cellulose-based separator was designed by fiber propionylation and silane crosslinking to reduce the side reactions aroused by hydroxyl groups and enhance the wet strength in liquid electrolytes to further improve its cycling properties. The optimal addition dosage of silane for the separator was investigated by characterization of its physiochemical and electrochemical properties. Finally, the action mechanism on the cycle performance of the separator was elucidated using molecular simulation and element analysis on the Li foil in a separator-assembled cell after cycling.

## 2. Materials and Methods

### 2.1. Materials

Bleached bagasse pulp was obtained from Guangxi Guitang (Group) Co., Ltd. (Guigang, China), which had a content of cellulose, hemicellulose and lignin of 80.51%, 18.36% and 0.11%, respectively. 3-Glycidyloxypropyltrimethoxysilane (GPTMS, 97%), pyridine and N,N-dimethylformamide (DMF) were purchased from Aladdin Biochemical Technology Co., Ltd. (Shanghai, China). Liquid electrolyte (1 M LiPF_6_ in ethylene carbonate (EC)/dimethyl carbonate (DMC)/diethyl carbonate (DEC) (1:1:1, *w*/*w*/*w*)), cathode (LiFeO_4_), and cell assembly materials were purchased from Zhuhai Saiwei Technology Co., Ltd. (Zhuhai, China).

### 2.2. Preparation of GPTMS-Crosslinked Propionylated Bagasse Fiber-Based Separator (PBF-GPTMS)

Propionylated cellulose fibers (PBFs) were synthesized according to our previous work [17]. GPTMS (1 wt%) was hydrolyzed in deionized water for 30 min. The obtained aqueous solution with the GPTMS amount of 0, 12 mg, 24 mg, 36 mg, and 48 mg, was added to the ethanol suspension (0.8 wt%, 30 mL) of PBFs (0.24 g, dry weight), respectively. The appropriate weight percent of added GPTMS was 0%, 5%, 10%, 15%, and 20% to that of the dry weight of the fibers, respectively. After stirring for 12 h at room temperature, the resulting fiber suspension was then poured into a polytetrafluoroethylene (PTFE) mold and dried at 40 °C overnight. The generated film was finally cured at 120 °C for 2 h. The obtained samples were named as PBF, PBF-GPTMS_5_, PBF-GPTMS_10_, PBF-GPTMS_15_, and PBF-GPTMS_20_, respectively. Additionally, the cellulose-based separator (BF) was prepared by the pristine bagasse fiber in the same way and used as a reference.

### 2.3. Characterization

ATR-FTIR (Tensor II, BRUKER, Munich, Germany), XPS (Thermo Scientific K-Alpha, MA, USA), and XRD (SMARTLAB, Tokyo, Japan) spectroscopy was employed to investigate the chemical structure and crystal properties, respectively. Surface morphology was characterized by SEM microscope (TESCAN MIRA LMS, Brno, Czech Republic). Degree of substitution (DS), tensile strength, thermal stability, thermal dimensional stability, porosity, and electrolyte uptake ratio were determined according to the literature [17]. The contact angle was obtained using a contact angle measuring instrument (DSA100, KRUSS, Hamburg, Germany). Ionic conductivity and electrochemical impedance spectroscopy were measured on an electrochemical working station (PGSTAT302N, Autolab, Herisau, Switzerland). The electrochemical window was measured in the assembled Li/separator/LiFeO_4_ cell. Rate capability and cycle performance of Li/separator/LiFeO_4_ cells were conducted on the battery testing system (LAND CT 2001A, Wuhan, China) at room temperature. The lithium plating/stripping test of symmetrical Li cells at a current density of 1 mA cm^−2^ was conducted.

### 2.4. Cell Assembly

The cathode slurry was prepared by mixing LiFePO_4_, carbon black, and polyvinylidene fluoride with a mass ratio of 90:5:5, respectively. The slurry was then coated on an aluminum foil with a loading amount of 1.5–2.0 mg cm^−2^ (1 C = 170 mAh g^−1^). The lithium metal coin cell (type: CR2032) was assembled by the sequence of negative case, shrapnel, gasket, Li anode, separator, LiFePO_4_ cathode, and positive case in a glove box filled with argon (O_2_ < 0.1 ppm, H_2_O < 0.1 ppm).

### 2.5. Molecular Simulation

In order to elucidate the effect of silane crosslinking on the wet strength of propionylated cellulose fibers, molecular simulation was conducted using the software of Materials Studio 17.1 [11]. Cellulose with a degree of polymerization of 10 was chosen as the model. The degree of substitution of the propionylated cellulose model was 1.10, while the grafting ratio of silane in the propionylated cellulose model was 0.20. The Forcite mechanical properties of the above models in a vacuum atmosphere and electrolyte solvents (EC, DMC, and DEC) were calculated in the COMPASS force field. In brief, the amount of solvent molecules was set to 60 in the mix model. The Anderson and Berendsen method was used for temperature and pressure control, respectively, in the process of energy optimization and molecular dynamics simulation according to the literature [18].

## 3. Results and Discussion

### 3.1. Design Idea

**Figure 1:**
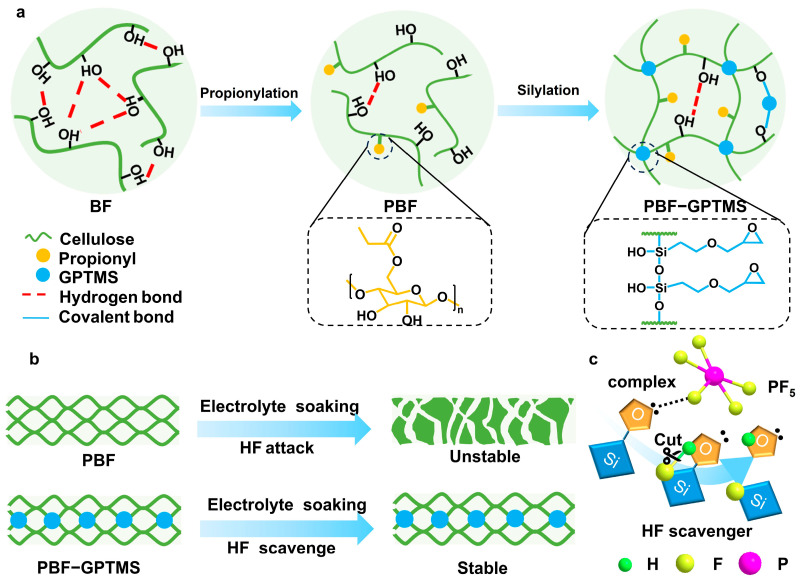
(**a**) Schematic illustration of the fabrication procedure of PBF-GPTMS separator, (**b**) working conditions of PBF and PBFGPTMS separators and (**c**) HF scavenging mechanism of GPTMS.

As shown in Figure 1, the preparation process of the PBF-GPTMS separator mainly involved two chemical reactions, including fiber propionylation and silane crosslinking. In the process of propionylation, the ester groups were introduced to the surface of the cellulose fibers, leading to fiber swelling in the carbonated electrolyte based on the “similarity-intermiscibility” principle. Although this phenomenon would endow the resulting separator with high electrolyte absorption and ionic conductivity, its low wet strength in the electrolyte would seriously affect the cycle performance of the assembled battery [17]. In order to solve this problem, silane was employed to crosslink the propionylated cellulose fibers. As a common crosslinking agent, GPTMS creates covalent bonding between the fibers, including C-O-Si and Si-O-Si bonds, which could improve the mechanical properties of cellulose-based materials [19]. In addition, it is known that the electrolyte molecule (LiPF_6_) would be hydrolyzed to produce PF_5_ and HF during battery working. The PF_5_ could be further hydrolyzed to form HF [20], which may promote corrosion of the battery. Thanks to the lone pair electrons in the formed Si-O-C structure in the PBF-GPTMS separator, HF could be scavenged to enhance the working lifespan of the assembled battery [15].

### 3.2. Chemical Structure and Morphology

**Figure 2:**
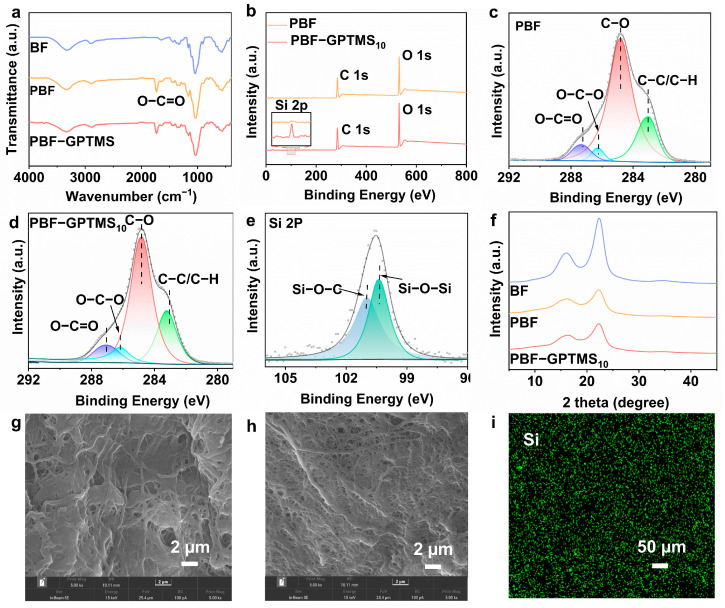
(**a**) FTIR spectra of BF, PBF, and PBFGPTMS_10_ separators; (**b**) XPS spectra and high-resolution C1s XPS deconvolution spectra of (**c**) PBF and (**d**) PBF-GPTMS_10_ separator; (**e**) high-resolution Si2p XPS deconvolution spectra of the PBF-GPTMS_10_ separator; (**f**) XRD patterns of BF, PBF, and PBF-GPTMS_10_ separators; SEM images of (**g**) PBF and (**h**) PBF-GPTMS_10_ separator; (**i**) EDS-mapping image of Si element on PBF-GPTMS_10_ separator.

The chemical structure of the PBF-GPTMS separator was investigated in detail. Figure 2a shows the ATR-FTIR spectra of the BF, PBF, and PBF-GPTMS_10_ separators. Compared with the BF separator, the PBF separator displayed a new peak at 1740 cm^−1^, which was attributed to the stretching vibration of the O-C=O groups [21]. This indicated the successful modification of the cellulose fibers. By titration, the degree of substitution (DS) value of the PBF was calculated to be 1.10 [22]. Further crosslinking by silane resulted in to PBF-GPTMS_10_ separator displaying no signal of the C-O-Si and Si-O-Si groups, which could be due to its coincidence with the C-O-C groups on the cellulose structure [23].

In order to further identify the chemical composition of the PBF-GPTMS_10_ separator, the XPS technique was employed and the according spectra are shown in Figure 2b. It can be seen that the PBF-GPTMS_10_ separator exhibited a new Si 2p peak at 101 eV in comparison with that of the PBF separator [24]. Moreover, the C 1s regions were deconvoluted to further understand the chemical structure of the separators (Figure 2c,d). As for the PBF separator, the relative peak area of the C-O structure was 86.31%, which decreased to 78.61% after silane crosslinking. Furthermore, the Si 2p deconvolution spectra of the PBF-GPTMS_10_ separator confirmed the chemical attachment of silane to the cellulose fibers via C-O-Si and Si-O-Si bonds (Figure 2e) [25].

Figure 2f shows the XRD patterns of the BF, PBF, and PBF-GPTMS_10_ separators. Two typical peaks were observed at 16.1° and 22.5° in the BF separator, which could be ascribed to the (101) and (002) lattice planes of cellulose I, respectively [26]. Its degree of crystallinity was determined to be 76.4% by Segal’s equation. Although the PBF and PBF-GPTMS_10_ separators displayed the same XRD patterns as the BF separator, their crystallinity was found to be 61.8% and 61.9%, respectively. This phenomenon may be attributed to the chemical reaction occurring in the crystalline phase of cellulose molecules during propionylation with pyridine as the catalyst [17]. The decreased crystallinity of the separators may be beneficial to ion transportation [27,28].

Apart from the chemical structure, the microstructure of the separators also plays a key role in their electrochemical properties. As shown in Figure 2g,h, the PBF-GPTMS separator exhibited a denser and more uniform pore structure in comparison with the PBF separator. Such morphology would endow the separator with higher mechanical strength and more homogenous deposition as well as the transportation of Li^+^ ions. In addition, the silicon element was evenly distributed on the surface of the PBF-GPTMS_10_ separator, which further confirmed the homogeneity of the separator (Figure 2i) [29].

### 3.3. Physicochemical Properties

**Figure 3:**
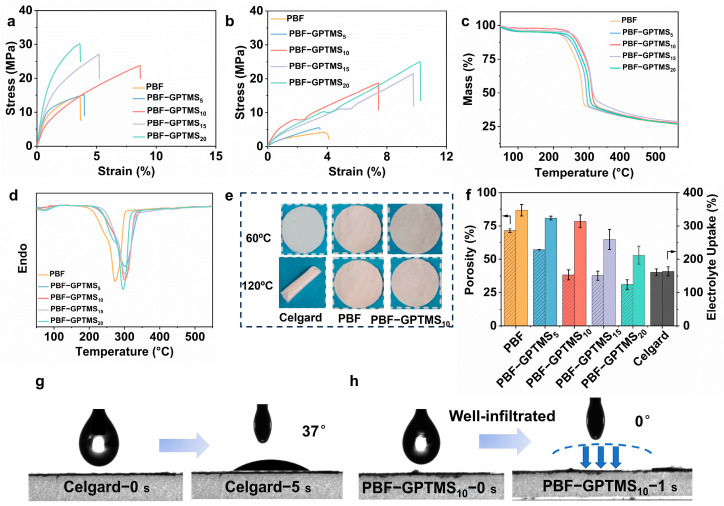
Tensile strength of PBF, PBF-GPTMS_5_, PBF-GPTMS_10_, PBF-GPTMS_15_, and PBF-GPTMS_20_ separators (**a**) before and (**b**) after soaked in the electrolyte; (**c**) TGA and (**d**) DTG curves of PBF, PBF-GPTMS_5_, PBF-GPTMS_10_, PBF-GPTMS_15_, PBF-GPTMS_20_ separators; (**e**) images of Celgard, PBF, and PBF-GPTMS_10_ separators at temperatures of 60 °C and 120 °C; (**f**) porosity and electrolyte uptake of PBF, PBF-GPTMS_5_, PBF-GPTMS_10_, PBF-GPTMS_15_, PBF-GPTMS_20_ separators; contact angle images of (**g**) Celgard and (**h**) PBF-GPTMS_10_ separators.

The good mechanical properties of the separators could prevent the internal short-circuit to promote the safety of the assembled LMBs [12]. As depicted in Figure 3a, the tensile strength of the PBF separator was 14.73 MPa. After silane blending, the generated PBF-GPTMS separators showed tensile strength from 15.10 MPa to 30.11 MPa with the increased doping content of GPTMS. This indicated the strength enhancement of the separator by silane crosslinking. Additionally, as the GPTMS content increased to 10%, the PBF-GPTMS_10_ separator displayed the highest elongation at a break of 9.10%. This could facilitate stress dissipation when the separator is subjected to external forces, thus preventing its premature fracture [30]. Further increasing the silane content leads to a decrease in the strain of the separator due to stress concentration [31].

Since the separator is immersed in the liquid electrolyte during the working of the battery, it is necessary to know its wet strength. As shown in Figure 3b, the tensile strength of the PBF separator was only 4.20 MPa after soaking in the electrolyte, which could be attributed to the swelling and softening effect of the electrolyte on the separator [10]. Instead, the wet strength of the PBF-GPTMS separators could still reach from 5.5 MPa to 25.0 MPa depending on the silane dosage. This could be ascribed to the covalent bonds between cellulose fibers and GPTMS, which would prevent electrolyte destruction on the fiber networks [32].

Additionally, the thermal stability of the separators is also an important parameter for the safety of LMB since heat is generated during the battery working to induce a thermal runaway. Figure 3c,d shows the TGA and DTG curves of the PBF and PBF-GPTMS separators. It can be seen that all the separators displayed similar mass loss between 215 and 330 °C due to the thermal decomposition of the cellulose molecules [33]. Furthermore, the main degradation peak (T_max_) of the PBF and PBF-GPTMS separators increased from 270 °C to 300 °C with the addition of GPTMS. This phenomenon could be due to the formation of a highly stable polysiloxane layer on the PBF-GPTMS separators [34]. Furthermore, the Celgard 2400 separator became curled after storage at 120 °C for 30 min, while the shape of PBF and PBF-GPTMS separators remained constant (Figure 3e). All these results indicated the good thermal stability of our proposed separators.

As displayed in Figure 3f, the porosity and electrolyte uptake of the PBF separator were 71.4% and 347.1%, respectively, which decreased to 28.31% and 212.2% with the increase in silane content. This could be explained by crosslinking causing a much denser network structure of the separator as shown in the SEM image [35,36]. Even though, the electrolyte absorption ratios of the PBF-GMTMS separators were still higher than that of the commercial Celgard separator (163.8%). In order to understand this phenomenon, the electrolyte wettability of the separators was investigated. As shown in Figure 3g, the electrolyte droplet hardly spread within a few seconds with a contact angle of 37° on the Celgard surface. Instead, the electrolyte was well-infiltrated into the PBF-GPTMS separator (Figure 3h). This confirmed that the PBF-GPTMS separator possessed excellent liquid electrolyte wettability, which could be due to the abundance of electrolyte-friendly groups, e.g., hydroxyl, ester, and epoxy groups [37].

### 3.4. Electrochemical Properties

**Figure 4:**
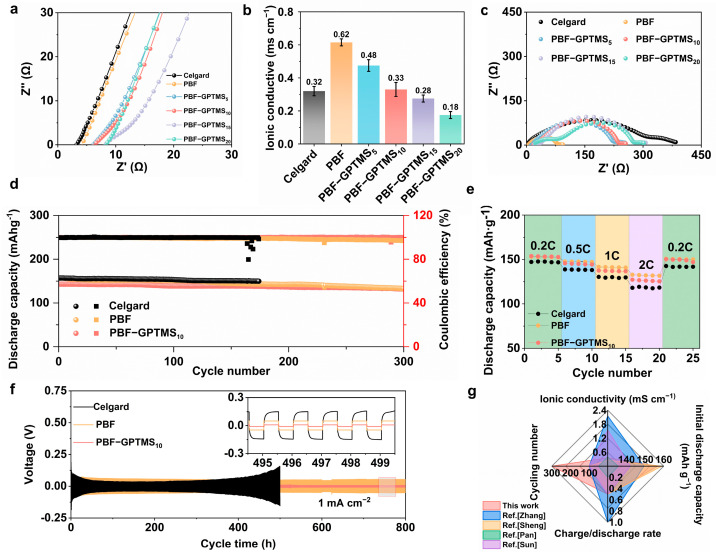
(**a**) Nyquist plots and (**b**) ionic conductivity of the Celgard, PBF, PBF-GPTMS_5_, PBF-GPTMS_10_, PBF-GPTMS_15_ and PBF-GPTMS_20_ separators at room temperature; (**c**) Nyquist plots of Li/separators/Li cells at room temperature; (**d**) cycle performance of LMBs assembled by Celgard, PBF, or PBF-GPTMS_10_ separators at 0.5 C; (**e**) C-rate performance of LMBs assembled by Celgard, PBF, or PBF-GPTMS_10_ separators at 0.2, 0.5, 1, 2, 3, and 5 C; (**f**) galvanostatic cycle curves of Celgard, PBF, and PBF-GPTMS_10_ separators at 1 mA cm^−2^; (**g**) radar diagram of comprehensive properties of our work and recently reported cellulose-based separators for LMBs.

Figure 4a displays the Nyquist plots of the PBF, PBF-GPTMS_5_, PBF-GPTMS_10_, PBF-GPTMS_15_, and PBF-GPTMS_20_ separators. Their bulk resistance was 4.6 ± 0.2, 5.9 ± 0.4, 7 ± 1, 8 ± 1, and 10 ± 1 Ω, and the corresponding ionic conductivity was calculated to be 0.62 ± 0.02, 0.48 ± 0.04, 0.33 ± 0.04, 0.28 ± 0.02, and 0.18 ± 0.02 mS cm^−1^ respectively (Figure 4b). The decreased ionic conductivity of the separators with the addition of silane could be due to their denser microstructure. When the silane dosage was no more than 10%, the PBF-GPTMS separator still had higher ionic conductivity than that of the Celgard separator (0.32 ± 0.03 mS cm^−1^) due to the better electrolyte wettability [38]. Additionally, the electrode/electrolyte interface also displayed a similar change tendency with the charge transfer resistance (R_ct_) increasing from 81.3 Ω to 280.6 Ω with an increase in the GPTMS content (Figure 4c). Even though, their properties were still better than that of the Celgard separator (302.3 Ω).

Figure S1 shows the linear sweep voltammetry (LSV) curves of separators. It can be seen that when the voltage rose to 3.5 V, the current in all the separator-assembled cells with LFP as the working electrode exhibited a sudden increase, which was consistent with the operating voltage of the LFP cathode [39]. Based on the above discussion, all the separators could be used under a voltage of at least 3.5 V, which made it possible for them to be applied in the LFP batteries. Since the PBF-GPTMS_10_ separator displayed the highest mechanical strength and ionic conductivity in the PBF-GPTMS separators, it was selected for the following cycle test of the assembled battery. Figure 4d shows the cycle performance of the assembled Li/separator/LiFePO_4_ battery at 0.5 C. The discharge capacity of the Celgard separator decayed from 147.5 to 99.8 mAh g^−1^ after 245 cycles. There was an irreversible short circuit in the PBF separator-assembled cell after 175 cycles. Instead, the initial discharge capacity of the PBF-GPTMS_10_ separator-assembled battery was 156.8 and 140.8 mAh g^−1^ with a capacity retention rate of 94.5% after 300 cycles and 93.6% after 160 cycles at 0.5 and 1 C, respectively (Figure S2). These cycling properties were better than those of the reported cellulose separators for LMBs (Figure 4g) [12,13,40,41], which could be due to the excellent wet strength and electrolyte absorption properties of the PBF-GPTMS_10_ separator.

Moreover, the rate performance of Li//LiFePO_4_ half-cells with different separators at different current densities was also investigated. As shown in Figure 4e, the Li/Celgard/LiFePO_4_ half-cell exhibited specific capacities of 147.2, 138.8, 130.4, 118.1, and 142.6 mAh g^−1^ at 0.2, 0.5, 1.0, 2.0, and 0.2 C, respectively. While, the corresponding data for the Li/PBF-GPTMS_10_/LiFePO_4_ half-cell were 153.9, 145.7, 137.5, 127.2, and 150.3 mAh g^−1^, respectively. It was clear that the PBF-GPTMS_10_ separator displayed better C-rate performance in comparison with that of Celgard due to its higher electrolyte uptake.

Finally, the long-term electrochemical stability of PBF-GPTMS against Li metal was demonstrated using symmetric Li/Li cells. As shown in Figure 4f, the cell with Celgard exhibited extremely high overpotential and unstable voltage profiles for 500 h at 1 mA cm^−2^. Its poor electrochemical behavior was caused by the nonhomogeneous deposition of Li^+^ ion due to the low electrolyte wettability, which further induced severe Li dendrites growth on the surface of the Li anode [42]. Although the PBF separator displayed a more stable overpotential than that of Celgard, its value was obviously higher than that of the PBF-GPTMS_10_ separator within 800 h. This could be explained by the high wet strength and HF scavenging of the PBF-GPTMS_10_ separator [43].

### 3.5. Working Mechanism

**Figure 5:**
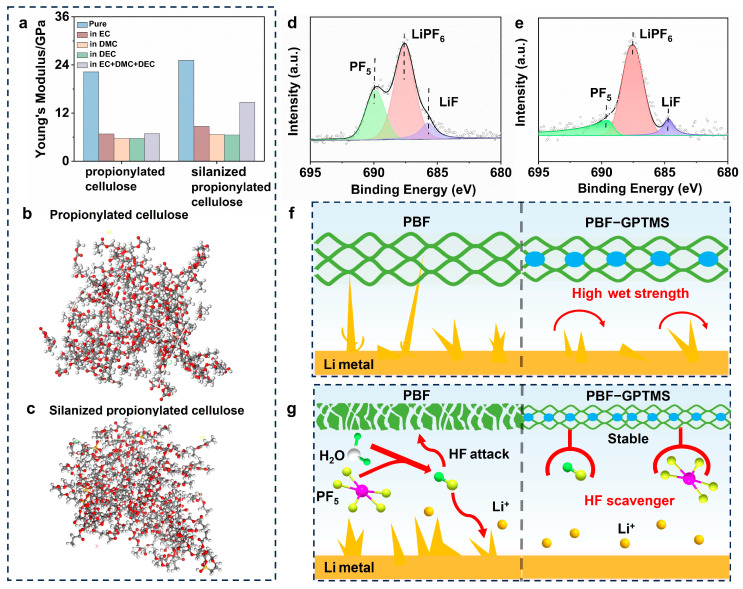
(**a**) Simulated Young’s modulus of molecular models under different environments, and (**b**) distinct details in the electrolyte solvent blended models: propionylated cellulose model and (**c**) silanized propionylated cellulose model; high-resolution F1s XPS deconvolution spectra of Li foil of the LMBs assembled by (**d**) Celgard or (**e**) PBF-GPTMS_10_ separators after 400 h at 1 C. Schematic diagram of the long cycle stability mechanism of the PBF-GPTMS separator: (**f**) high strength and (**g**) HF scavenger.

To further understand the mechanism of the long lifespan of the PBF-GPTMS separator, the effect of electrolyte solvents (EC, DMC, and DEC) on the propionylated cellulose model before and after silane grafting was analyzed by molecular simulation. As shown in Figure 5a, Young’s modulus of the silanized propionylated cellulose model was 25.17 and 14.64 GPa in vacuum/pure and EC/DMC/DEC conditions, respectively, which was higher than that of the propionylated cellulose model (22.27 and 6.89 GPa). Figure 5b,c presents the distinct details in the corresponding blended models. It can be seen that the silanized propionylated cellulose model was more stable in the solvent compared with the propionylated cellulose model. These results confirmed the strength enhancement of the propionylated cellulose by silane crosslinking. This could ensure the dimensional stability of the PBF-GPTMS_10_ separator during battery cycling.

In addition to the wet strength of the separator, a side reaction occurs during the battery work, which would also affect its cycle life [27]. Accordingly, the chemical composition of the Li foil in the assembled cell after cycling was examined using XPS spectroscopy. There were three peaks at 689.9, 687.4, and 684.8 eV in the F 1s XPS spectra of the cycled Li foil (Figure 5d,e), which were attributed to the signals of PF_5_, LiPF_6_, and LiF, respectively [44]. It was obvious that the Li foil in the PBF-GPTMS_10_-assembled cell displayed a lower PF_5_ content (17.19%) in comparison with that in the Celgard-assembled cell (30.47%). This could be attributed to that the lone pair electrons in the oxygen atoms in the Si-O motif of the PBF-GPTMS separator were able to effectively form a complex with PF_5_ [45]. In this case, a lower amount of HF would be generated, which might alleviate the corrosion on the separator. Based on the above discussion, it could be concluded that silane crosslinking endowed the PBF-GPTMS separator with both wet strength improvement and HF scavenger to further enhance its cycling performance as illustrated in Figure 5f,g.

## 4. Conclusions

In this study, a long-life cellulose-based separator was fabricated by GPTMS crosslinking of the propionylated cellulose fibers. The resulting PBF-GPTMS separator exhibited a wet tensile strength of 25.00 MPa, which was 5.95 times higher than that of the PBF separator. Due to the complexation between the lone pair electrons in the oxygen atoms of the Si-O motif in the separator and PF_5_, a lower amount of HF would be generated to reduce side reactions during battery working. Accordingly, the LMBs assembled with the PBF-GPTMS separator exhibited excellent cycle performance with a capacity retention of 94.5% and 93.6%, respectively, after 300 cycles at 0.5 C and 160 cycles at 1 C.

## Data Availability

The data presented in this study are available on request from the corresponding author.

## Supplementary Materials

The following supporting information can be downloaded at: https://www.mdpi.com/article/10.3390/polym17091203/s1, Figure S1: LSV curves of Celgard, PBF, PBF-GPTMS_5_, PBF-GPTMS_10_, PBF-GPTMS_15_ and PBF-GPTMS_20_ separators with LFP as the working electrode at a scan rate of 5 mV s^−1^; Figure S2: Cycle performance of LMB assembled by PBF-GPTMS_10_ at 1 C.
