# Supplementary material for: A Silane Cross-Linked Cellulose-Based Separator for Long-Life Lithium Metal Batteries Application
Source: Polymers (Basel). 2025 Apr 28;17(9):1203. doi: 10.3390/polym17091203 (PMC12073682; doi:10.3390/polym17091203)
Supplement: Supplementary file 1 [file polymers-17-01203-s001.zip › polymers-3593214-supplementary.pdf]

Supplementary data

Silane cross-linked cellulose-based separator for long-life lithium metal batteries application

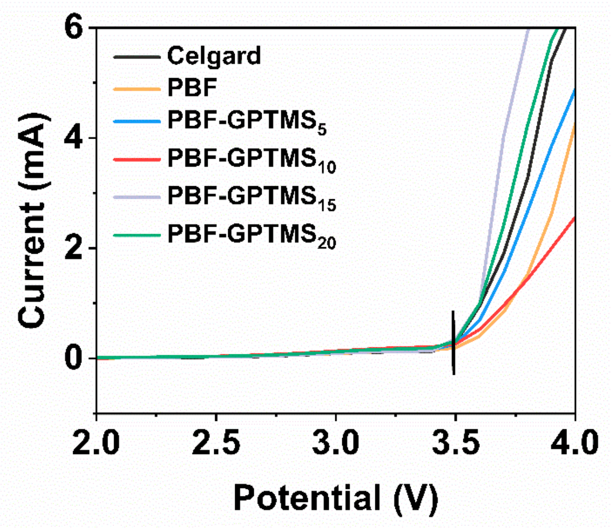

Figure. S1 LSV curves of Celgard, PBF, $PBF-GPTMS_5$, $PBF-GPTMS_{10}$, $PBF-GPTMS_{15}$ and $PBF-GPTMS_{20}$ separators with LFP as the working electrode at a scan rate of 5 mV s^{-1}.

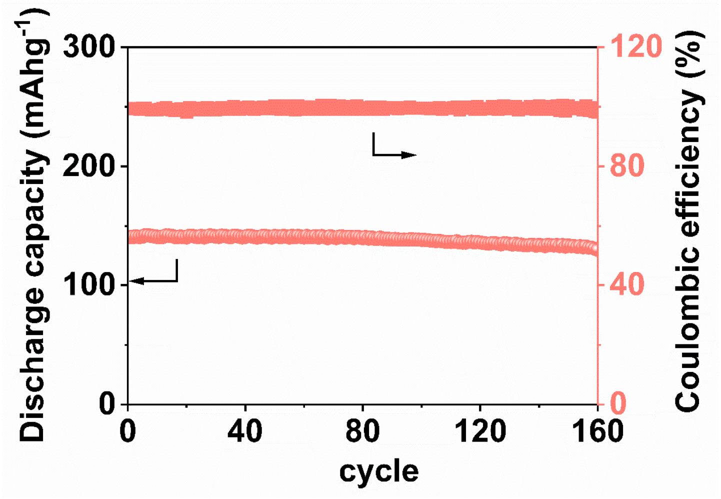

Figure. S2 Cycle performance of LMB assembled by $PBF-GPTMS_{10}$ at 1 C.
